# Changing the Paradigm in Public Health and Disability through a Knowledge Translation Center

**DOI:** 10.3390/ijerph15020328

**Published:** 2018-02-13

**Authors:** Kerri A. Vanderbom, Yochai Eisenberg, Allison H. Tubbs, Teneasha Washington, Alex X. Martínez, Amy Rauworth

**Affiliations:** 1 Department of Physical Therapy, University of Alabama at Birmingham/Lakeshore Research Collaborative, Birmingham, AL 35209, USA; 2 Department of Disability and Human Development, University of Illinois at Chicago, Chicago, IL 60608, USA; yeisen2@uic.edu; 3 The National Center on Health, Physical Activity and Disability, Birmingham, AL 35209, USA; allisonh@lakeshore.org (A.H.T.); alexm@lakeshore.org (A.X.M.); amyr@lakeshore.org (A.R.); 4 Department of Health Behavior, University of Alabama at Birmingham/Lakeshore Research Collaborative, Birmingham, AL 35209, USA; teneasha@uab.edu

**Keywords:** disability inclusion, social determinants, health inequity

## Abstract

People with disabilities are a health disparity population that face many barriers to health promotion opportunities in their communities. Inclusion in public health initiatives is a critical approach to address the health disparities that people with disabilities experience. The National Center on Health, Physical Activity and Disability (NCHPAD) is tackling health disparities in the areas of physical activity, healthy nutrition, and healthy weight management. Using the NCHPAD Knowledge Adaptation, Translation, and Scale-up Framework, NCHPAD is systematically facilitating, monitoring, and evaluating inclusive programmatic, policy, systems, and environmental (PPSE) changes in communities and organizations at a local and national level. Through examples we will highlight the importance of adapting knowledge, facilitating uptake, developing strategic partnerships and building community capacity that ultimately creates sustainable, inclusive change.

## 1. Introduction

### People with Disabilities Face Health Inequities

People with disabilities are an unrecognized disparity population [1] that experience health inequities rooted in a history of systematic discrimination and exclusion [2]. The Americans with Disabilities Act defines disability as a person who has a physical or mental impairment that substantially limits one or more major life activities [3]. While having a disability does not equate to being unhealthy, individuals with disabilities experience a greater incidence of poor health than other underserved groups and people without disability [4]. For instance, 41% of individuals with disabilities are obese, compared to only 25.2% of their non-disabled peers aged 18 and over [5]. Subsequently, the cost of disability among US adults was estimated to be nearly $400 billion USD, with health care costs accounting for nearly 27% of all health care expenditures [6]. As the largest minority group in the United States, the health status of individuals with disabilities is a major and significant public health concern.

Public health practitioners seeking to reduce health inequities among racial and ethnic minorities have focused on programmatic, policy, systems and environmental (PPSE) strategies. In fact, much of the work funded by the Centers for Disease Control and Prevention (CDC) uses this approach [7]. However, the PPSE strategies implemented in communities across the United States will not necessarily reduce health inequities for people with disabilities due to the pervasive exclusion of people with disability embedded in the social determinants of health “where people live, work, and play” [8]. Exclusion is perpetuated through the many barriers that people with disabilities face in their communities. Barriers are created, for example, by a lack of transportation [9], inaccessible facilities and community environments [10] and discrimination [11]. These barriers can affect access to healthcare [12,13,14], participation in community and social activities with friends, family, and neighbors [15,16], schools [17,18,19], and employment [20,21,22]. An important solution to address these barriers and effectively reduce health disparities is for practitioners to implement adapted PPSEs, to ensure unequivocal inclusion of people with disabilities. Inclusion is based on social justice principles and occurs when all community members are presumed competent; are recruited and welcome as valued members of their community; fully participate and learn with their peers; and experience reciprocal social relationships [23]. The purpose of this article is to describe how the National Center on Health, Physical Activity and Disability (NCHPAD) facilitates the adaptation and implementation of inclusive PPSE.

## 2. Methods

NCHPAD focuses on facilitating inclusive PPSE changes in local and national initiatives in the areas of physical activity, healthy nutrition, and healthy weight management. To carry out their work, NCHPAD has developed and uses a knowledge-to-practice framework to systematically build and advance the evidence-base of PPSEs inclusive of people with disabilities [24]. In a previous article by Rimmer and Vanderbom, NCHPAD’s framework NCHPAD Knowledge Adaptation, Translation, and Scale-up (N-KATS) was discussed [24]. That article was specific to physical therapists and how they can support people with disabilities transitioning from rehabilitation to the community using the N-KATS. The present article describes the N-KATS as it is being used more broadly to increase inclusion of people with disabilities in public health initiatives.

**Figure 1:**
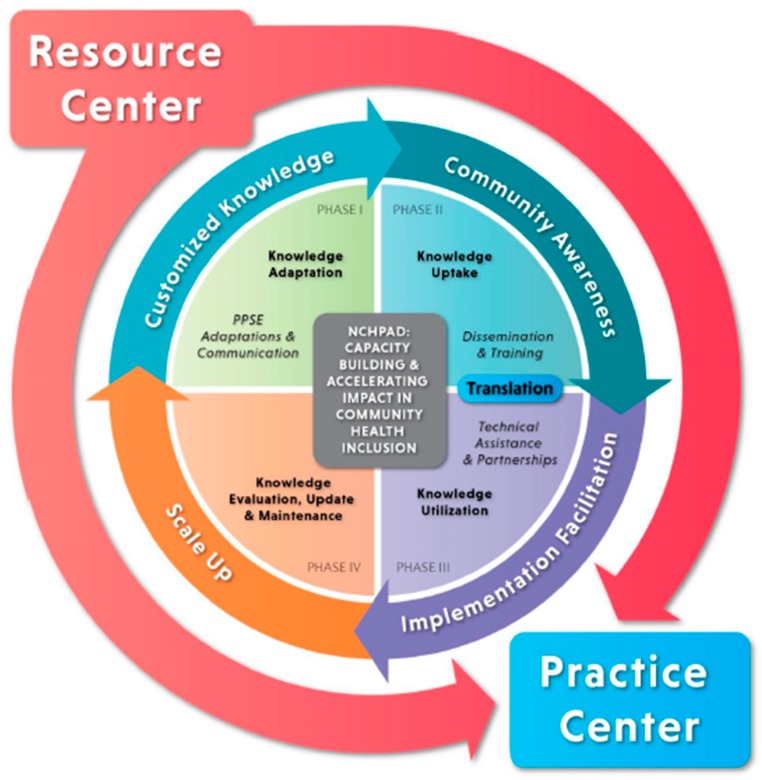
The National Center on Health, Physical Activity, and Disability Knowledge Adaptation, Translation, and Scale-up Framework.

The N-KATS is comprised of four phases: Phase 1—new and existing evidence- and practice-based knowledge is collected and adapted for the local context (i.e., community, organization); Phase 2—customized resources are disseminated to strategic partners and key stakeholders with appropriate training and tools; Phase 3—NCHPAD Expert Inclusion Specialists (EISs) serve as facilitators by providing technical assistance to strategic partners and key stakeholders implementing inclusion strategies (used to adapt PPSEs); Phase 4—evaluation is conducted and successful elements of practice (e.g., inclusion strategies) are archived and scaled to other organizations and communities [24] (Figure 1). The N-KATS framework supports inclusive PPSEs at local and national levels by helping professionals serve as knowledge brokers and inclusion facilitators [24] in five sectors: (1) public health professionals; (2) fitness, recreation, and sport; (3) healthcare providers; (4) disability and aging service providers and (5) educators. A NCPHAD EIS, based on their experience and expertise, is assigned to one of the five targeted sectors. The EIS then works within each sequential phase, focusing on their specific sector. An advisory panel made up of individuals with disabilities and family/caregivers and national experts from the different sectors, provide guidance and review of the work conducted through the NKATS phases. Each step is explained in more detail in the following sections.

### 2.1. Phase 1: Knowledge Adaptation: PPSE Adaptation and Communication

Ideally, health professionals would create inclusive and accessible PPSEs at the development stage. However, the vast majority of evidence-based health promotion research unintentionally or unknowingly excludes people with disabilities [25]; in fact, disability is often used as an exclusion criteria, therefore limiting the generalizability of findings to people with disabilities [26]. Consequently, many PPSEs are not developed with the intention of including people with disabilities and this underrepresented population continues to be excluded from participation in health promotion opportunities that could improve/maintain their health status. Adapting evidence-based PPSEs for people with disabilities holds great potential for accelerating the use of existing and new evidence-based findings in this population [27].

To address this issue, NCHPAD EISs adapt both PPSEs and they develop and adapt a wide array of tailored resources and tools to support the inclusion of people with disabilities. There is a push–pull dynamic where organizations reach out to NCHPAD and request help to make adaptations, or NCHPAD identifies evidence-based PPSEs to adapt and develop resources needed to implement them and disseminates them to communities. The EIS who oversees each sector is responsible for researching inclusion and accessibility needs and then makes the adaptations, or creates the inclusion resource content to address the needs of that sector. In order to identify and prioritize which evidence-based PPSEs to adapt, specific criteria are used: (1) level of evidence (i.e., research tested, practice tested, or emerging practice) [28] designated to the program by the database where it was located(e.g., Researched Tested Intervention Programs, Center on Training and Research Translation (TRT), Healthy People 2020 Evidence-Based Resources); (2) current usage (i.e., national/state, private/public organizations, no longer used, not known); (3) whether a program is freely available or has costs associated; (4) whether adaptations are requested by an organization (yes/no); and (5) agreement of the needs or importance of the PPSE by advisory panel members. Using the prioritization criteria, NCHPAD is able to strategically choose to focus on the adaptation PPSEs that will significantly impact health equity for people with disabilities.

Once a PPSE has been identified to be adapted, the NCHPAD EIS use a systematic, evidence-informed method that evolved from the ADAPTE process [29]. The ADAPTE method was developed to adapt evidence-based guidelines developed for one population, to then be used in a different population [29]. The steps in the process comprise of set-up, adaptation, and then the final review. To describe the adaptation process, the example of adapting the Girls on the Run program will be used. Girls on the Run is a positive youth (i.e., third through eighth-grade girls) development program with a curriculum designed to teach life skills through physical activity [30].

During the set-up step, the EIS must acquire either a hard or electronic copy of the program being adapted. NCHPAD acquires the program directly from the organization, or if it is a freely available program, then it is obtained online. Next, the people or organizations who should be involved are identified and invited to be a part of the adaptation team. Those invited may include the organization who developed the program, specific advisory panel members, and others who are experts in the area (e.g., adapted physical education/activity), and/or individuals with specific disabilities or their family members/caregivers. The adaptation team members have roles assigned to them which vary from assisting with the search for resources/information that help inform what adaptations should be made, assisting with developing the adaptations, reviewing and commenting on the adaptations made, and endorsing the final product(s).

Depending on whether it is a policy, program, system, or environment adaptation, the adaptation process may be relatively quick, or it may take a few months. For instance, making inclusive changes to one Girls on the Run policy may not take as long as making inclusive changes to the Girls on the Run curriculum. Further, the NCHPAD EIS engages in conversations with the organization curriculum developers to ensure adaptations are made without compromising the fidelity of the program’s key components.

There are two main tools that the EIS uses to adapt PPSEs. The Guidelines, Recommendations and Adaptations Including Disability (GRAIDs), are inclusion strategies used to adapt PPSEs in physical activity, nutrition, and the community social and physical environments [27]. A second tool, the GRAIDs Domain Framework, takes an ecological approach and consists of five core domains: the built environment, equipment and technology, services, instructional, and policy [27]. The five domains are applied to the PPSE at different points during the adaptation process. By addressing each of the five domains, organizations can be sure that they will be providing an inclusive policy, program, system, or environment, and at the same time increase the likelihood that inclusion will be sustained and supported by organizational policies.

The adaptation process starts with an initial review of the program for disability inclusion. Using the Girls on the Run example, the EIS applies the GRAIDs Domain Framework to the overall program to identify the specific areas where adaptations are needed. For instance, a route used for running during a Girls on the Run activity may be partially on a road with potholes and large rocks; this may necessitate an adaptation in the built environment domain if there is a participant who has balance issues or uses a wheelchair. After the inclusion gaps have been identified, the specific adaptations needed to address the gaps are chosen or developed. The adaptations are chosen from the GRAIDs (existing inclusion strategies), or developed using a triangulation of grey or white literature, an advisory panel, and disability stakeholder feedback. An example adaptation from the GRAIDs used to address the issue of the rough road is to simply re-route the activity to an area where the road is well paved or there is a sidewalk.

Finally, once the adaptations have been compiled and either added to the original program manual or a supplemental inclusion manual is created, there is an internal and external review before the program is finalized. The GRAIDs Domain Framework may be used again by reviewers to examine the adapted program for thorough disability inclusion. The internal review is conducted by another NCHPAD EIS, staff person, or a local partner who gives edits and feedback. The external review is then conducted by two advisory panel members. Adaptations are discussed during a conference call to ensure that both reviewers had a similar concept or understanding of the adaptation. If reviewers do not reach agreements during the meetings, the EIS improves, creates and submits a new adaptation until the reviewers reach agreement. If the reviewers agree on all adaptations, then the adapted program can move forward to the next step towards dissemination. Depending on the level of involvement by the organization that developed the original program, they may also review the adapted program, give feedback, and endorse it. The NCHPAD EIS carefully documents the adaptation resources and process in an online database so that it is replicable and/or can be improved upon.

### 2.2. Phase 2: Knowledge Uptake: Dissemination and Training

NCHPAD’s active approach to getting information and resources into the hands of targeted audiences is based on three broad goals of dissemination [31]: (1) to increase the reach of the inclusion message and resources; (2) to increase an organization’s motivation to use and apply inclusion principles and strategies; and (3) to increase an organization’s ability to use and implement the inclusion principles and strategies. The Agency for Healthcare Research and Quality’s (AHRQ) 10 dissemination steps [32] are used to direct NCHPAD’s dissemination efforts (Table 1).

Knowing the target audiences and identifying which modes of dissemination are most effective for each target audience are essential parts of dissemination and advertising the availability of the specific, adapted PPSEs. For each PPSE or resource that the NCHPAD EISs develop or adapt, consideration is given to ensure that it is tailored to the target audience: how will it be used? Is the content relevant and is the correct language/terminology used for the target audience? What format should it be in (e.g., brochure, infographic, newsletter, web-based, etc.)? Who will help spread the message about the product/resource? What resources does NCHPAD have for dissemination and promotion?

NCHPAD uses multiple methods for dissemination for each of the five target sectors that include a tailored newsletter, videos, and website resources. The American Association on Health and Disability (AAHD) and the National Association of County and City Officials (NACCHO) are NCHPAD’s dissemination partners that work alongside NCHPAD to spread the inclusion resources and information. They are knowledgeable organizations with connections to help NCHPAD reach the different target audiences, as well as help identify other organizations that potentially could help with dissemination. In addition, both dissemination partners give feedback and recommendations regarding the format and messaging most effective for specific sectors.

Through AAHD, NCHPAD is able to disseminate materials and information to disability-focused organizations/associations and stakeholders; through NACCHO NCHPAD is able to spread their inclusion materials to public health departments around the country that may not be including people with disability in their current PPSEs, or who need more guidance about how to improve their efforts with their local public health initiatives.

Other dissemination methods used are presenting at conferences/webinars and maintaining an active social media presence. The development and use of a dissemination plan helps to increase the likelihood that the materials and information will be taken up and used by the target audiences in order to increase the inclusion of people with disabilities in PPSEs. To measure the success of dissemination efforts, NCHPAD monitors the reach of materials disseminated, as well as gathering feedback through surveys about presentations and other information in order to constantly improve content and dissemination methods.

Training is conducted with both strategic partners and other organizations or groups that make a request. Training is delivered in-person or through an online platform and may last one hour or one and a half days. Broad training topics include, but are not limited to disability awareness and legislation; building healthy, inclusive, communities; inclusion and accessibility assessments of programs, organizations, and the built environment; and guidelines on disability inclusion in physical activity and nutrition. Social justice is a crucial foundational aspect of all NCHPAD training and teaches trainees the importance of including participants with disabilities and their family members/caregivers or disability representing/advocating organizations. To continue the Girls on the Run example, training topics and resources for the local councils and their community coaches might include basic disability awareness/knowledge, how to assess and adapt activities and environments to be inclusive and accessible, and then specific training on inclusion and their curriculum.

### 2.3. Phase 3—Knowledge Utilization: Technical Assistance and Partnerships

#### 2.3.1. Facilitating Top-Down Implementation

Once the adapted knowledge has been developed and disseminated, NCHPAD helps strategic partners implement the inclusive PPSEs. Strategic partnerships at both a local and national level are vital in order to act upon the social determinants of health [33]. Building community capacity by sharing resources, knowledge, and forming networks, as in Phase 2, is an effective method to provide services and promote health [34]. These active, sustained networks of partners are key in order to address the complex factors that influence health behaviors and outcomes for people with disabilities [34]. However, to promote action on and the use of the resources and knowledge disseminated, NCHPAD works with partners to help facilitate their uptake of adapted, inclusive PPSEs and resources.

NCHPAD partners with national organizations that have broad reach through local affiliates. For example, the National Recreation and Parks Association (NRPA) partnered with NCHPAD to develop and implement the Parks for Inclusion Initiative. NRPA is a non-profit organization dedicated to the advancement of public parks, recreation, and conservation and has a membership of 60,000 park and recreation professionals and advocates [35]. To improve inclusion at NRPA, NCHPAD tailored inclusion guidelines and other resources for the recreation and parks services. As local agencies begin to use these resources, NCHPAD will provide technical assistance to the local park and recreation agencies, and work through the NRPA to collect data on how materials supported the uptake of inclusive practices of local affiliates. Results will be disseminated and best practices will be scaled-up across the field of parks and recreation.

#### 2.3.2. Facilitating Bottom-Up Implementation

**Figure 2:**
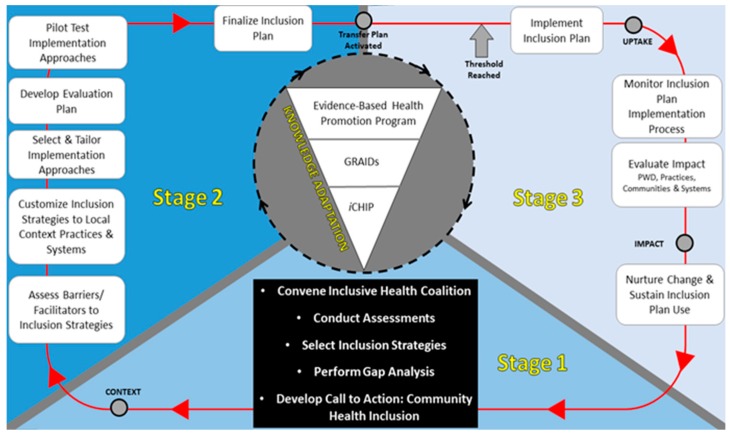
NCHPAD inclusive CAN-IMPLEMENT Process.

To facilitate the practice of inclusion in organizations and communities, NCHPAD utilizes an adapted version of CAN-IMPLEMENT, an evidence-based model that was developed to support the implementation of evidence-based guidelines [36]. The NCHPAD iCAN-IMPLEMENT (inclusive CAN-IMPLEMENT) Process [24] (Figure 2) consists of three stages and multiple steps within each stage to guide the use of inclusion strategies in communities and organizations. The stages are: (1) Identifying the specific inclusion problem(s); (2) Developing the solution; and (3) Implementation, evaluation, and sustaining inclusive practices. The process and outcomes documented by partners who use the NCHPAD iCAN-IMPLEMENT process will help build the evidence-base around inclusion practices.

Forming collaborative strategic partnerships at a local and national level is a necessary step in the process of making inclusive, sustainable changes. The aim of these partnerships is to help facilitate the needs of people with disabilities in the development of public health initiatives. Without the explicit inclusion of people with disabilities in the public health agendas, the problem of exclusion in PPSEs and health disparities in this population will be perpetuated. Utilizing a systematic evidence-based method for implementing inclusion, such as the NCHPAD iCAN-IMPLEMENT process, NCHPAD is able to document what works and what does not work, in relation to inclusive practices. These results will help develop the inclusion science needed to guide and support communities and organizations to continue this work in the future.

### 2.4. Phase 4—Knowledge Evaluation, Update, and Maintenance

NCHPAD’s activities are systematically evaluated to capture both the successes and challenges that organizations and communities experience when implementing adapted, inclusive, health-promoting PPSEs. NCHPAD’s evaluation team uses the socio-ecological model and measures the different levels of national partners (top-down) and local communities (bottom-up) to evaluate if the inclusive PPSEs have been successful. For instance, working with national partners, such as Girls on the Run, NCHPAD measures how knowledge of inclusion has changed as a result of training for national and regional staff. NCHPAD then works with Girls on the Run to monitor adaptations used by coaches and track the participation of girls with disability across hundreds of local communities. At the community level, NCHPAD partners first assess current PPSEs using the Community Health Inclusion Index [37] to identify areas of inclusion need. This baseline data can also be used to monitor changes over time. Finally, organizations using NCHPAD-adapted PPSEs or resources measure success through the increased participation of people with disability in community settings, and when applicable or able, improvements in health. At the programmatic level, the identification of participation of people with disabilities involves advising organizations to add one or two questions that serve as disability identifiers, such as the six disability questions used in the American Community Survey [38].

The evaluation team also works with organizations and communities to monitor the implementation process and includes things such as the inclusion of people with disabilities and their family members/caregivers in the decision making at the local level and whether or not steps were completed in the NCHPAD iCAN-IMPLEMENT process. Measuring these components gives insight into why an adapted program or an individual inclusion strategy was or was not successful; and the steps where there was breakdown can be identified and addressed.

The successful strategies identified through NCHPAD’s comprehensive evaluation will be scaled-up and spread to other organizations and communities; the less successful elements can be altered or discarded. The evaluation team documents the strategies and then they are incorporated into a master database of inclusion strategies. The successful strategies used by organizations and communities will be shared on a website to be accessed by others. NCHPAD will make results of this work available once information has been collected and analyzed.

## 3. Conclusions

As a national health promotion resource for people with disabilities, NCHPAD is in a unique situation to advance the practice of inclusive, health-promoting PPSEs. Maintaining a focus on improving access to and the inclusion of community physical activity, nutrition, and weight management programs and services, NCHPAD facilitates inclusive PPSEs through adaptation, dissemination, implementation facilitation, and scale-up methods. Working beyond silos and developing strategic partnerships, building community capacity, and ultimately creating sustainable change at the local, state, and national levels are critical approaches to achieve the ultimate goal of improving the health outcomes of people with disabilities. NCHPAD aims to create a culture of health [39] that is inclusive of people with disabilities and disrupts the history of exclusion in public health.

## Figures and Tables

**Table 1:** The Agency for Healthcare Research and Quality’s 10 Dissemination Strategies [32] Utilized by the National Center on Health, Physical Activity, and Disability.

1. Plan from the outset for promotion and dissemination of inclusion materials and information created and adapted
2. Identify the target audience as early as possible
3. Engage those who can help learn about and reach the target audiences
4. Use the insights of social marketing to help craft messages about the inclusion materials or information, as well as the dissemination activities
5. Be strategic about the timing of the release of the inclusion materials
6. Be strategic about the positioning of important information within the inclusion materials disseminated
7. Actively work with the media, locally and nationally, to promote inclusion materials and information
8. Use advertising to promote the inclusion materials and information
9. Use outreach to promote the inclusion materials and information and facilitate their use
10. Gather and analyze feedback on the dissemination efforts
